# TRAJECTORIES OF HEALTH RECOVERY AFTER HIP FRACTURE IN OLDER ADULTS: A SCOPING REVIEW

**DOI:** 10.1093/geroni/igac059.3013

**Published:** 2022-12-20

**Authors:** Tingzhong (Michelle) Xue, Jiepin Cao, Leila Ledbetter, Margaret Graton, Yan Zhan, Dingyue (Demy) Wang, Youran Lee, Eleanor McConnell

**Affiliations:** Duke University School of Nursing, Durham, North Carolina, United States; NYU Grossman School of Medicine, New York City, New York, United States; Duke University, Durham, North Carolina, United States; Duke University, Durham, North Carolina, United States; Yale University, Orange, Connecticut, United States; Duke University, Durham, North Carolina, United States; Duke University, Durham, North Carolina, United States; Duke University, Durham, North Carolina, United States

## Abstract

Hip fracture recovery outcomes in older adults are characterized by high mortality, lowered functional status, and feelings of being disrupted from a normal life. Studying recovery trajectories through the lens of resilience can provide novel perspectives for developing interventions targeting to promote recovery. However, the lack of knowledge of recovery trajectories and their variations in hip fracture patients impedes such efforts. This review aims to synthesize current evidence on how multiple health domains change longitudinally after hip fracture in older adults. The Joanna Briggs Institute scoping review methodology was followed, and seven databases were searched including Medline (PubMed), EMBASE, Web of Science Core Collection, CINAHL, Proquest Dissertations and Theses, Cochrane Central Register of Controlled Trials, and Cochrane Database of Systematic Reviews. No date limits were applied, and the final search resulted in 7,515 articles. Articles in English with participants aged 60 years and above who experienced a low-energy, nonpharmacological hip fracture in any health setting were selected. Results regarding multiple domains of health outcomes will be synthesized, including physical health (e.g. functional status, pain, nutrition, and mobility/physical performance), cognition, psychosocial health (e.g. depression, anxiety, social isolation, loneliness, and behavioral and psychological symptoms of dementia when individuals with dementia were included), and multidimensional outcomes such as health-related quality of life. Methodological challenges and limitations will be discussed. This review has important implications for clinicians and researchers to improve individualized treatment plans and research methodologies by providing a comprehensive, critical review of knowledge regarding health trajectories in older adults after hip fracture.

